# NUTRIC score use around the world: a systematic review

**DOI:** 10.5935/0103-507X.20190061

**Published:** 2019

**Authors:** Audrey Machado dos Reis, Ana Valéria Gonçalves Fructhenicht, Luis Fernando Moreira

**Affiliations:** 1 Programa de Pós-Graduação em Alimentação, Nutrição e Saúde, Faculdade de Medicina, Universidade Federal do Rio Grande do Sul - Porto Alegre (RS), Brasil.; 2 Programa de Pós-Graduação em Cirurgia, Faculdade de Medicina, Universidade Federal do Rio Grande do Sul - Porto Alegre (RS), Brasil.

**Keywords:** Malnutrition, Nutritional status, Nutrition assessment, Critical illness, Intensive care units

## Abstract

**Objective:**

To collect data on the use of The Nutrition Risk in Critically Ill (NUTRIC) score.

**Methods:**

A systematic literature search was conducted in accordance with the Preferred Reporting Items for Systematic Reviews and Meta-Analyses (PRISMA) statement. Reviews, abstracts, dissertations, protocols and case reports were excluded from this review; to be included in the review, studies needed to specifically evaluate the NUTRIC score and to have been published in English, Spanish or Portuguese.

**Results:**

We included 12 (0.8%) studies from our search in this review. Ten studies (83.3%) were observational, 1 was a pilot study (8.3%) and 1 was a randomized control trial (8.3%). All of the included studies (100%) chose not to use IL-6 and considered a high nutritional risk cutoff point ≥ 5. There were 11 (91.7%) English language studies *versus* 1 (8.3%) Spanish language study. Mechanical ventilation and a high NUTRIC score were significantly correlated in four studies. The association between intensive care unit or hospital length of stay and nutritional high risk was significant in three studies. Seven studies found a statistically significant association between the NUTRIC score and mortality.

**Conclusion:**

The NUTRIC score is related to clinical outcomes, such as length of hospital stay, and is appropriate for use in critically ill patients in intensive care units.

## INTRODUCTION

Malnutrition is common in hospitalized patients and highly prevalent in the population of critically ill individuals.^(1,2)^ Malnutrition is associated with increased morbidity, mortality, occurrence of nosocomial infections, prolonged hospitalization, worse functional status at discharge from intensive care units (ICU) and increased hospital costs.^(3,4)^

Most of the tools used to assess nutritional risk include a variety of criteria to identify nutritional risk, such as food/nutritional intake, physical examination, severity of illness, anthropometric data and functional assessment.^(5)^ Many of these criteria are difficult to obtain in critically ill patients because almost all of these patients require mechanical ventilation (MV) and sedation.^(5)^ Changes in weight can be influenced by fluid status, given the large volumes necessary to maintain hemodynamic stability.^(5)^ Many traditional tools do not provide information regarding inflammatory status, which is crucial in critically ill patients because it is one of the factors responsible for hypermetabolic status and muscle wasting.^(5)^

In 2011, Heyland et al. presented a new screening tool called Nutrition Risk in Critically Ill (NUTRIC) score, which was validated for ICU patients.^(6)^ This score evaluates adverse outcome risk (mortality, MV) modifiable by intensive nutritional intervention.^(6)^ The variables incorporated in this score are: age, Acute Physiology and Chronic Health disease Classification System II (APACHE II) score, Sequential Organ Failure Assessment (SOFA) score, comorbidities, days in the hospital prior to admission to the ICU and Interleukin-6 (IL-6).^(6)^ Proposed in 2016, a modified NUTRIC without IL-6 can be used considering a high nutritional risk cutoff point ≥ 5.^(7)^

The purpose of this review is to collect data on the use of the NUTRIC score.

## METHODS

A systematic literature search was conducted in accordance with the Preferred Reporting Items for Systematic Reviews and Meta-Analyses (PRISMA) statement^(8)^ in December 2017. The search was carried out in four databases: Medical Literature Analysis and Retrieval System Online (MEDLINE), Latin American and Caribbean Health Sciences Literature (LILACS), Scientific Electronic Library Online (SciELO) and Cochrane Collaboration. The search strategy for these databases were defined by terms related to NUTRIC [NUTRIC, Nutrition Risk in Critically Ill score] and terms related to nutritional assessment [nutritional risk, nutritional status] in addition to "critical illness". The terms were enclosed in quotation marks, and the search operators "and" and "or" were used. Reviews, abstracts, studies protocols, dissertations and case reports were excluded from this review.

**Figure 1:**
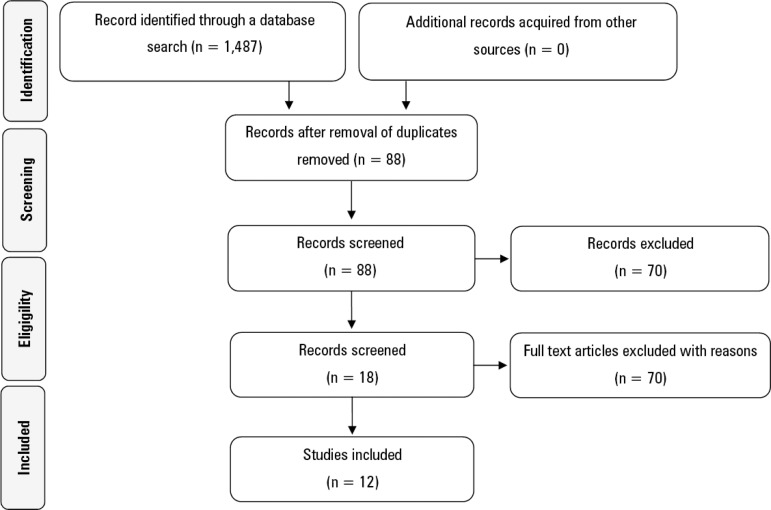
Flowchart of eligibility.

Moreover, to be included in the review, studies needed to specifically evaluate the NUTRIC score and to have been published in English, Spanish or Portuguese. Finally, articles were screened according to the following steps: at first, duplicates were excluded. Then, the remaining articles were screened by title, abstract and text in full. Articles were selected based on the eligibility criteria as outlined above. If eligibility could not be determined during the initial screening of the title and abstract, full-text articles were accessed to determine inclusion. Both the study selection and data extraction were concurrently performed by two of the authors (AR and AF). If there was any doubt regarding the eligibility criteria, a third evaluator (LFM), another author, made the final decision. MEDLINE, LILACS, Cochrane and SciELO provided 1189, 30, 179 and 89 articles, respectively. More details are shown in figure 1.

## RESULTS

Out of 1487 studies, 12 (0.8%) were included in this review.^(7,9-19)^ All of the included studies (100%) evaluated adults.^(7,9-19)^ Ten studies (83.3%) had an observational design,^(7,9-12,14-19)^ 1 was a pilot study (8.3%)^(13)^ and 1 was a randomized control trial (8.3%).^(7)^ All of the included studies (100%) chose not to use IL-6.^(7,9-19)^ There were 11 studies (91.7%) in English^(10-19)^ *versus* 1 study (8.3%) in Spanish.^(9)^ More details and the main results are presented in table 1 and figure 1.

**Table 1:** Study details

Author, Country	Study	Sample	Exclusion criteria	APACHE II; SOFA (points)	Age; Gender (M)
Rahman et al.^(7)^ Canada	Randomized control trial.	1199 MV patients with multi-organ failure and expected length of stay > 5 days.	None	Not available	Not available
Moretti et al.^(9)^ Argentina	Prospective observational study.	368 patients aged ≥ 18 years old and MV within 24 hours of ICU admission.	Patients whose data could not be collected.	20.7; 7.7	52 (18 - 93); (68%)
Lee et al.^(10)^ Malaysia	Prospective observational study.	154 patients aged ≥ 18 years old, and VM within 48 hours and in ICU > 72 hours.	Patients moribund, readmitted, or transferred from another ICU.	26.9; 12.4	51.29 ± 15.73; 83 (54%)
Mendes et al.^(11)^ Portugal	Prospective observational multicenter study.	1143 patients aged ≥ 18 years old and in ICU > 72 hours.	Patients with brain dead or readmitted, or transferred from another ICU.	20; 7	64 (51 - 75); 740 (65%)
Mukhopadhyay et al.^(12)^ Singapore	Prospective observational study.	401 patients aged ≥ 18 years old and in ICU > 24 hours.	Patients discharged or died within 24 hours.	27.3; 9.5	60.0 ± 16.3; (62%)
Rosa et al.^(13)^ Brazil	Pilot study.	50 patients in ICU > 48 hours.	Not available.	18.5; 5	61.4 ± 15.3; 26 (52%)
Kalaiselvan et al.^(14)^ Indian	Prospective observational study.	687 patients aged ≥ 18 years old in ICU and MV > 48 hours.	Readmitted, or transferred from another ICU.	22.2; 6.7	55.7 ± 17.5; 458 (68%)
Coltman et al.^(15)^ United States	Prospective observational study.	139 patients aged ≥ 18 years.	Unable to communicate in English.	13; 2.7	59.0 ± 16.4; 146 (50%)
Özbilgin et al.^(16)^ Turkey	Prospective descriptive and cross-sectional study.	152 postoperative patients aged ≥18 years.	Psychiatric disorder patients, difficulty cooperating, nutrition history not available, vomiting, taking appetite-enhancing medications, and pregnant or breastfeeding.	13.5; 3.1	Not available
de Vries et al.^(17)^ The Netherlands	Retrospective study.	475 patients aged ≥ 18 years, requiring (non)-invasive VM within 24 hours.	Time between ICU admission and discharge < 24 hours, data incomplete, or pregnancy.	22; 8	71 (57 - 81); 215 (45%)
Lew et al.^(18)^ Singapore	Prospective observational study.	439 patients aged ≥18 years in ICU ≥ 24 hours.	Patients with inaccessible data.	24.5; 8.6	61.4 ± 15.8; 259 (59%)
Compher et al.^(19)^ Canada	Prospective observational study.	2,853 MV patients at least 4 ICU days.	Patients with very short LOS or expectation of imminent demise.	Not available	> 4 ICU days: 61.2 (17.3); 1739 (60.9%) > 12 ICU days: 59.7 (17.4); 1003 (62.5%)

APACHE II - Acute Physiology and Chronic Health Evaluation II; SOFA - Sequential Organ Failure Assessment; ICU - intensive care unit; MV - mechanical ventilation; LOS - length of hospitalization.

### NUTRIC applicability

In Brazil, a pilot study was conducted.^(13)^ Portuguese translation and adaptation were required to validate the NUTRIC score for use in Brazil.^(13)^ The authors evaluated 50 individuals whose data were easily obtained from medical records, and neither nutritionists nor physicians reported difficulties in registering them.^(13)^ All five healthcare professionals who participated in the pilot study reported that the new version of the NUTRIC score was easy and clear to understand as well as practical and fast to apply.^(13)^

### Altered NUTRIC

Moretti et al. conducted a study that used C-reactive protein (CRP) instead of IL-6 (NUTRIC-2, ≥ 6 points) *versus* no inflammatory marker (NUTRIC-1, ≥ 5 points).^(9)^

NUTRIC-2 used cut-off value of ≥ 6 points to define high risk, as suggested by Heyland et al.,^(6)^ and had a sensitivity and specificity of 37.76% and 88.95%, respectively.^(9)^ A cut-off value of 3 points led to a sensitivity close to 70% and a specificity of 60%.^(9)^ However, the sensitivity and specificity of the area under the receiver operator characteristic (ROC) curve were lower for predicting mortality than the original study (0.671 and 0.679 *versus* 0.783, respectively).^(6,9)^

### NUTRIC and high nutritional risk

In all but one of the included studies, patients were classified as having a high nutritional risk if the NUTRIC score was ≥ 5 points.^(9-17)^ The Moretti group used a cutoff ≥ 6 for the NUTRIC score with CRP.^(9)^ In this study, a high nutritional risk was found in 93 (25%) patients.^(9)^

More than half (55.8%) of 203 patients were at high nutritional risk in the Lee et al. study.^(10)^ Similar values were found by Mendes et al. in Portugal (48%),^(11)^ Rosa et al. in Brazil (46%),^(13)^ Kalaiselvan et al. in India (42.5%),^(14)^ and Mukhopadhyay et al. (53.8%)^(12)^^)^ and Lew et al. (67.9%) in Singapore.^(18)^

A minor percentage of patients were at high nutritional risk in the studies performed by Coltman et al. in the United States of America (26%)^(15)^ and Özbilgin et al. in Turkey (22.4%).^(16)^

### NUTRIC and mechanical ventilation

Özbilgin et al. demonstrated no relationship between the NUTRIC score and MV use (p = 0.136) or MV time (p = 0.245).^(16)^ Lew et al. did not associate high risk with MV time (2.0 [1.0 - 4.3] *versus* 2.0 [1.0 - 5.0], p > 0.050).^(18)^ Kalaiselvan et al. did not find an association between high nutritional risk and MV-free days (2 [± 2.8] *versus* 1.7 [± 1.9], p = 0.100).^(14)^

On the other hand, de Vries et al. included only ventilated patients in their study.^(17)^ They found that the median duration of ventilation was significantly increased in patients with a high NUTRIC score (+ 2.5 days, p < 0.001).^(17)^ Moretti et al. also only included ventilated patients.^(9)^ They demonstrated an association between NUTRIC-1 (without IL-6) and NUTRIC-2 (version with CRP) with MV days in surviving patients (p = 0.034 and p = 0.010, respectively).^(9)^

In the study by Mukhopadhyay et al., for 273 patients who received MV, significant differences were noted between high and low nutritional risk in terms of MV duration (3.3 [1.5 - 5.7] *versus* 3.5 [2.0 - 7.0], p < 0.001).^(12)^ Mendes et al. performed a logistic regression analysis and found that the NUTRIC score was associated with fewer MV-free days (odds ratio - OR 1.46; 95% confidence interval - 95%CI 1.16 - 1.85; p = 0.002; n = 1,124).^(11)^

### NUTRIC and complications

Three studies analyzed complications.^(9,15,16)^ Additional rehabilitation after discharge was more associated with high nutritional risk compared to no risk (13% *versus* 10%).^(15)^ Özbilgin et al. found a significative relationship between pulmonary complications and a high NUTRIC score (p = 0.030).^(16)^

Moretti et al. found that the mean of score of patients with pneumonia compared with those without pneumonia was 3.19 (± 1.58) *versus* 3.77 (± 1.96) points for NUTRIC-1 (with no inflammatory marker) (p = 0.034) and 3.62 (± 1.69) *versus* 4.16 (± 2.06) for NUTRIC-2 (with CRP) (p = 0.054), respectively.^(9)^

### NUTRIC length of hospitalization

The association between hospital length of stay (LOS) and the NUTRIC score was not significant in only one study (p = 0.134).^(16)^

Coltman et al. identified that patients at high nutritional risk had the longest ICU and hospital LOS compared to those with no risk (hospital LOS, 6.9 [± 6.7] *versus* 12.1 [± 10.7] days; ICU LOS, 3.7 [± 3.5] *versus* 5.4 [± 5.3]).^(15)^

Other studies found significant associations between high nutritional risk and ICU LOS: Kalaiselvan et al. 9.0 (± 4.2) *versus* 7.8 (± 5.8) (p < 0.010);^(14)^ Mendes et al. 10.0 (5.0 - 16.5) *versus* 8 (5.0 - 14.0) (p < 0.001);^(11)^ and Mukhopadhyay et al. 5.0 (3.0 - 9.0) *versus* 3.5 (2.0 - 7.0) (p < 0.010).^(12)^

Length of stay was also significantly shorter by 5.1% for each 10% increase in protein intake relative to goal in high-risk patients at 4 days (p = 0.010) and 12 days (p = 0.002) and for each 10% increase in energy intake (4 days; p = 0.019) and (12 days; p = 0.002).^(19)^

### NUTRIC and mortality

Ten studies analyzed the relationship between the NUTRIC score and mortality.^(7,9-12,14-17, 19)^ Rahman et al. estimated that mortality was increased by 1.4 times for every point increase of the NUTRIC score.^(7)^ Higher NUTRIC scores were significantly associated with higher 6-month mortality (p < 0.001).^(7)^

Coltman et al. demonstrated that high-risk patients had highest rates of death compared to those with no risk (14.0% *versus* 3.0%).^(15)^ Other authors found statistically significant associations: Kalaiselvan et al. 41.4% *versus* 26.1% (p < 0.001);^(14)^ Mukhopadhyay et al 36% *versus* 12.7% in MV patients (p < 0.001);^(12)^ and Lew et al. 9.2% *versus* 39.3% (p < 0.001).^(18)^ Other groups associated a high NUTRIC score with death, including Moretti et al. (NUTRIC-1, 4.23 [± 1.92] *versus* 3.06 [± 1.72], p < 0.001) and (NUTRIC-2, 4.68 [± 1.98] *versus* 3.39 [± 1.83], p < 0.001);^(9)^ Özbilgin et al. (5.0 [± 2.03] *versus* 3.17 [± 1.46], p = 0.002);^(16)^ and de Vries et al. (6.0 [5.0 - 7.0] *versus* 5.0 [3.0 - 6.0], p < 0.001).^(17)^ Mendes et al. showed that a high nutritional risk increased the risk of mortality (OR 3.84; 95%CI 2.80 - 5.26; p < 0.001; n = 1122).^(11)^

In Compher et al., mortality of high-risk patients was significantly decreased by 6.6% (p = 0.003) and 10.1% (p = 0.003) at 4 days and 12 days, respectively, with each 10% increase in protein intake relative to goal.^(19)^ The same was observed for each 10% increase in energy intake (4 days and 12 days; p < 0.001).^(19)^

In addition, for Lee et al., among patients with a low nutrition risk, 60-day mortality was increased by approximately 6 times in the group that received the diet prescribed ≥ 2/3 compared with < 2/3 (OR 6.30; 95%CI 1.17 - 33.78; p = 0.032).^(10)^ Among patients at high nutrition risk, no difference in mortality status was found.^(10)^

## DISCUSSION

This systematic review showed that many patients are at high nutritional risk on ICU admission. We also demonstrated that the NUTRIC score is becoming increasingly popular around the world. Application of the NUTRIC score in patients at the beginning of hospitalization in this sector has become relevant, and it is associated with MV, clinical complications, hospitalization time and death.

The NUTRIC score was validated by Heyland et al. and is the first nutritional risk assessment tool developed specifically for ICU patients that can identify patients at nutritional risk.^(6)^ Heyland et al. considered the need for a more specific nutritional risk evaluation tool for ICU patients and found that inquiring about weight loss and their nutritional situation was insufficient, mainly due to the heterogeneous nature of ICU patients.^(6)^ Thus, they incorporated different variables into the score (age, APACHE II, SOFA, comorbidities, days at hospital before ICU and IL-6).^(6)^ Later, Rahman et al. validated the modified NUTRIC, which allows the exclusion of IL-6 levels, if not available, to assess nutritional risk at admission.^(7)^

The NUTRIC score is based on a conceptual model designed around how to measure acute and chronic inflammation.^(6)^ The importance of inflammation and illness severity are well recognized in the characterization of malnutrition,^(1)^ such as its association with hospital length of stay.^(20)^ Patients with a higher score have worse clinical outcomes, such as high mortality rates.^(16)^

There are no traditional markers of nutritional risk, such as body mass index (BMI), weight loss, oral intake, or physical assessment, and the NUTRIC score only considers the severity of illness.^(6)^ However, in the original study regarding validation, data such as BMI, percentage oral intake in the prior week, and percentage weight loss in the past three months were not associated with mortality.^(5)^

Early identification of individuals at nutritional risk who may benefit from nutritional therapy is paramount in the hospital environment, including the ICU setting.^(6)^ Heyland et al. considered that greater awareness of nutritional risk assessment tools, such as the NUTRIC score, and risk factors, such as BMI and duration of ICU stay, may enhance the delivery of calories and protein to patients who need them the most.^(6)^ Although many instruments have indicated that all critically ill patients are at nutritional risk due to their clinical conditions,^(21,22)^ they may not have the same risk of adverse events related to malnutrition.^(6)^

The NUTRIC score shows the importance of developing specific scores for individuals with particular clinical conditions.^(13)^ Additionally, the NUTRIC score is a fast, practical instrument that can be incorporated into the routine care of ICUs.^(13)^ One clear advantage of the NUTRIC score is its applicability in situations in which patients are unable to respond verbally, as in MV, since the variables used in this scoring system are objectively obtained from data routinely registered in patients' medical records.^(13)^

Regarding the future perspectives for NUTRIC, its use is promising for health professionals. The use of IL-6 in the score makes it difficult to use because no study has included it. We must value studies that seek to simplify NUTRIC with variables that are more commonly available, such as CRP inclusion.^(9)^ We found that there are many observational studies relating the NUTRIC score to unfavorable clinical outcomes, but only one group has performed an interventionist study.^(7)^ Therefore, is necessary to conduct studies that show the relationship of clinical outcomes through NUTRIC intervention. In addition, it is important that its use is not limited to nutritionist as the NUTRIC score is capable of pointing out relevant clinical outcomes, such as complications and death.

This was the first systematic review of the use of the NUTRIC score. The number of studies that evaluated the performance or application of the score is relatively low because of its recent validation. We consider this a limitation of our review.

## CONCLUSION

The NUTRIC score is related to clinical outcomes, length of hospital stay and death and is appropriate for use in critically ill patients in intensive care units. More studies are needed to evaluate this tool for this particular population.

## Figures and Tables

**Table 2:** Main results

Author, Country	Main results
Rahman et al.^(7)^ Canada	Mortality at 28 days was multiplied by 1.4 for every point increase of the NUTRIC score. There is a strong positive association between nutritional adequacy and 28-day survival in patients with a high NUTRIC score, but this association decreases with the decreasing NUTRIC score. Higher NUTRIC scores are also significantly associated with higher 6-month mortality (p < 0.001).
Moretti et al.^(9)^ Argentine	Mortality increased in relation to the score (p < 0.001). The mean CRP was higher in mortality (p = 0.001) and VM time (p = 0.010), and the AUC increased in a similar way to IL-6 in the original work (0.008 and 0.007, respectively).
Lee et al.^(10)^ Malaysia	For patients with low nutritional risk, mortality was increased by approximately 6 times in the group that received ≥ 2/3 of prescribed than both < 2/3 (p = 0.032).
Mendes et al.^(11)^ Portugal	A high NUTRIC score was associated with longer hospitalization (p < 0.001), fewer days free of MV (p = 0.002) and higher 28-day mortality (p < 0.001).
Mukhopadhyay et al.^(12)^ Singapore	The NUTRIC score (p < 0.001) was associated with 28-day mortality.
Rosa et al.^(13)^ 2016 Brazil	The Portuguese version was easily introduced into four Brazilian ICUs, and the prevalence of patients with a high score was 46%.
Kalaiselvan et al.^(14)^ Indian	NUTRIC score (p < 0.001), use of vasopressor drug (p < 0.005) and BMI (p < 0.002) were associated with 28-day mortality. In 273 patients who received MV, significant differences were noted between the high and low NUTRIC groups in terms of mortality (p < 0.001), ICU LOS (p < 0.014), and duration of MV (p < 0.001).
Coltman et al.^(15)^ United States	Patients determined to be at nutritional risk using the NUTRIC score alone or in combination with any other tool had the highest rates of death. A larger proportion of patients requiring additional rehabilitation after discharge was seen with NUTRIC score. Patients identified as being at nutritional risk or malnourished using NUTRIC had the longest hospital LOS and ICU LOS.
Özbilgin et al.^(16)^ Turkey	There was a positive correlation with mortality and the NUTRIC score (p=0.020) and pulmonary complications (p = 0.030).
de Vries et al.^(17)^ The Netherlands	The discriminative ability of the NUTRIC score for 28-day mortality is (ROC-AUC) 0.768 (95% CI 0.722 - 0.814) with an associated LR+ of 1.73 (95% CI 1.53 - 1.95) and LR− of 0.24 (95% CI 0.14 - 0.39) when comparing low with high (> 4) scores.
Lew et al.^(18)^ Singapore	High NUTRIC score was associated with hospital mortality (p < 0.001).
Compher et al.^(19)^ Canada	In high-risk but not low-risk patients, mortality was lower with greater protein (4-d sample: p = 0.003; 12-d sample: p = 0.003) and energy (4-d sample: p < 0.001; 12-d sample: p < 0.001) intake. In high-risk but not low-risk patients, time to discharge alive was shorter with greater protein (4-d sample: p = 0.010; 12-d sample: p = 0.002) and energy intake (4-d sample: p = 0.020; 12-d sample: p = 0.002).

NUTRIC - Nutrition Risk in the Critically Ill; CRP - C-reactive protein; MV - mechanical ventilation; AUC - area under the curve; IL - interleukin; ICU - intensive care unit; BMI - body mass index; LOS - length of stay; ROC - receiver operating characteristic; LR - likelihood; d - day; 95%CI - 95% confidence interval.
